# Invasive *Acer negundo *outperforms native species in non-limiting resource environments due to its higher phenotypic plasticity

**DOI:** 10.1186/1472-6785-11-28

**Published:** 2011-11-24

**Authors:** Annabel J Porté, Laurent J Lamarque, Christopher J Lortie, Richard Michalet, Sylvain Delzon

**Affiliations:** 1 UMR 1202 Biodiversité Gènes et Communautés, Université de Bordeaux, Talence, 33400, France; 2 UMR 1202 Biodiversité Gènes et Communautés, Institut National de la Recherche Agronomique, Cestas, 33610, France; 3 Department of Biology, York University, Toronto, M3J 1P3, Canada

## Abstract

**Background:**

To identify the determinants of invasiveness, comparisons of traits of invasive and native species are commonly performed. Invasiveness is generally linked to higher values of reproductive, physiological and growth-related traits of the invasives relative to the natives in the introduced range. Phenotypic plasticity of these traits has also been cited to increase the success of invasive species but has been little studied in invasive tree species. In a greenhouse experiment, we compared ecophysiological traits between an invasive species to Europe, *Acer negundo*, and early- and late-successional co-occurring native species, under different light, nutrient availability and disturbance regimes. We also compared species of the same species groups *in situ*, in riparian forests.

**Results:**

Under non-limiting resources, *A. negundo *seedlings showed higher growth rates than the native species. However, *A. negundo *displayed equivalent or lower photosynthetic capacities and nitrogen content per unit leaf area compared to the native species; these findings were observed both on the seedlings in the greenhouse experiment and on adult trees *in situ*. These physiological traits were mostly conservative along the different light, nutrient and disturbance environments. Overall, under non-limiting light and nutrient conditions, specific leaf area and total leaf area of *A. negundo *were substantially larger. The invasive species presented a higher plasticity in allocation to foliage and therefore in growth with increasing nutrient and light availability relative to the native species.

**Conclusions:**

The higher level of plasticity of the invasive species in foliage allocation in response to light and nutrient availability induced a better growth in non-limiting resource environments. These results give us more elements on the invasiveness of *A. negundo *and suggest that such behaviour could explain the ability of *A. negundo *to outperform native tree species, contributes to its spread in European resource-rich riparian forests and impedes its establishment under closed-canopy hardwood forests.

## Background

Plant invasions, a main component of global change, are a source of agricultural and economic problems worldwide but also a major ecological threat for biodiversity [1-3], which makes it crucial to understand the key mechanisms that can lead to invasions in an ecosystem. Recent studies concluded that plant invasions are the result of complex interactions between the exotic species performances (i.e., invasiveness), the recipient environment's vulnerability (i.e., invasibility) and the history of the introductions (see for instance [4,5]). With regard to species invasiveness, the success of invasive species seemed to be largely due to their superiority over native species in terms of growth rate and spread into recipient ecosystems; this superiority seemed related to higher values of traits related to fitness such as growth rate, maturity age, fecundity and seed dispersal [4,6-8]. Invasive tree species are doing a lot of damage worldwide [9], and a recent meta-analysis [10] reported that growth rate is a key determinant of the success of invasive tree species. Furthermore, comparative studies that measured native versus invasive tree growth have shown that invasive species are associated with higher growth rates than natives [11-15]. Hence, a reasonable starting point for understanding the dynamics of tree invasion is to precisely quantify growth rate of invasive species in contrast to natives.

In most cases, a higher growth rate results from a more efficient resource use. Major traits related to resource use include leaf traits such as Specific Leaf Area (SLA) or Total Leaf Area (TLA) that serve as a surrogate for light use and carbon assimilation [16] or physiological traits such as photosynthetic rates or nitrogen leaf content [16]. Higher SLA often correlates with a growth advantage for exotic tree species over native ones [13,15,17]. A recent comparison of 29 invasive and non-invasive pine species [18] showed that invasiveness could be predicted by using only species growth rate and SLA. On the other hand, it was also demonstrated that invasive tree species were characterised by higher photosynthetic rates compared to native ones [19,20]. The same conclusion was presented on two species of the genus *Acer *(*A*. *platanoides *vs. *A. saccharum*, [12]).

However, it is not only their superior morphological or physiological traits that could confer a competitive advantage to invasive species relative to natives but also the dynamic response of their traits [21]. Invasiveness can indeed be related to a higher plasticity of the plant traits in response to environmental changes [22]. Phenotypic plasticity defined as the ability of organisms to alter their morphology and/ or physiology in response to varying environmental conditions has thus been cited to increase the success of invasive species [23-26] since it increases their realised ecological niches. In general, phenotypic plasticity has been applied to the study of plant invasions through the following two distinct hypotheses [27]: (1) invasive species are more plastic than exotic non-invasive species or native species of the recipient communities [28-30] and/ or (2) invasive populations of exotic species have evolved and present a greater plasticity relative to native populations [30-33]. Hence, it is important to compare phenotypic plasticity amongst related pairs of invasive and native species [21] as well as amongst exotic species with different degree of invasive success [26,34,35]. Relative differences in the mean value of traits associated to their plastic response to a range of environmental conditions can provide a powerful tool to explore the invasiveness of exotic species and thus provide mechanistic explanations of invasion events.

To date, most plant invasion studies have focused on herbaceous species. However, although many of the world's most serious invasive plant species are woody species such as several Pine species [36,37], very few studies have explored the link between plasticity and invasiveness in invasive tree species [30,38]. Consequently, empirical studies on tree species are critical to identify the general role of plasticity in explaining invasiveness [21]. Box elder maple (*Acer negundo*) native to North America has been widely planted as an ornamental tree species throughout central and southern Europe. Recently, it has colonised riparian habitats in many regions spreading at the expense of native species and leading to monospecific stands [39-43] in particular in South-Western France [44]. To determine whether resource use efficiency contributes to *A. negundo *invasiveness, we compared its growth and related morphological and physiological traits to that of native co-occurring tree species: *Fraxinus excelsior*, *Fraxinus angustifolia*, *Populus nigra*, *Alnus glutinosa *and *Salix alba*. We used greenhouse treatments spanning different light regimes, soil nutrient resources and disturbance levels. Additionally, adult trees in different riparian forests were compared *in situ *to ensure that results obtained on seedlings under artificial environments were relevant. Specifically, three main questions were addressed here: (i) Are there any growth differences between the invasive *Acer negundo *and native species? (ii) Which traits could best explain the success of the invasive species? (iii) Do the studied species present any plasticity and differences in magnitude of plasticity amongst the environmental conditions?

## Results

### Growth rate

**Figure 1:**
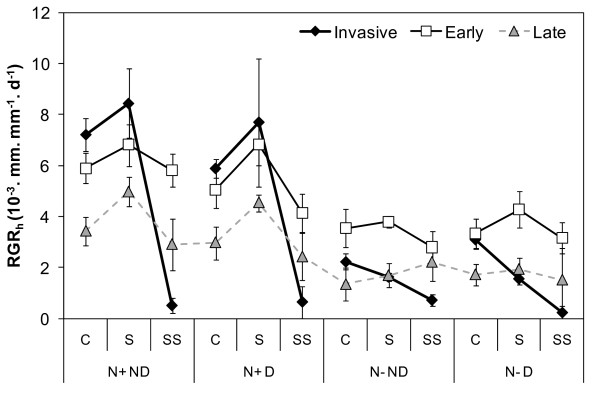
**Relative height growth rates of the invasive and native species according to the environmental conditions**. Relative height growth rates (RGR_h_, mm. mm^-1^. d^-1^. 10^-3^). Values are means of nine to twelve seedlings (± 1 SE of the mean) for the invasive species (*A. negundo*, full diamonds), late-successional native species (*F. excelsior *and *F. angustifolia*, grey triangles) and early-successional native species (*S. alba *and *P. nigra*, open squares) across the three shade levels (Full light C, Shade S, Deep shade SS), the two nutrient levels (nutrient supply N+ vs. no supply N-) and the two disturbance regimes (Disturbed D vs. Non-disturbed ND).

Figure 1 presents the relative growth rate responses to light level, nutrient availability and disturbance as applied to the native and invasive tree seedlings. Nutrient availability induced the most significant difference in growth rate whatever the species: the relative height growth rates (RGR_h _values) were 3.2 (p = 0.0013), 2.0 (p = 0.0013) and 1.6 (p < 0.0001) times higher in fertilised compared to non-fertilised treatments, for the invasive, late-successional and early-successional species, respectively (table 1). Disturbance did not induce any significant difference in growth rate whatever the species and whatever the shade or fertilisation levels. On the other hand, the response to light varied amongst species. There was no significant effect of the shade treatment on the RGR_h _of neither group of native species. On the contrary, the shade treatment (p = 0.0116) and the interaction shade*fertilisation (p = 0.0155) had a significant impact on the relative growth rate of the invasive species. Under fertilised and full light conditions, *A. negundo *and early-successional native species displayed significantly higher RGR_h _than late-successional native species (with 50 to 110% increases according to the treatment); in constrast, under fertilised and deep shade conditions, *A. negundo *presented dramatically lowered growth rates relative to the early-successional species. To sum up, the growth rate plasticity in response to resource (light × nutrient) availability was 9.6 times higher in *A. negundo *seedlings relative to the native seedlings: *A. negundo *growth rate was 13 times higher in full light and shade (on average) compared to the deep shade level (Figure 1) under high nutrient availability, whereas the same environmental changes only resulted in a 1.23 and 1.5 time increase in RGR_h _for the early- and late-successional native species, respectively.

**Table 1:** Split -split- plot analysis of variance of tested environmental conditions for measured traits and group of species.

Variables	Species	Shade	Fertilization	Disturbance	S×F	S×D	F×D
RGR_h_	Invasive	**0.012**	**0.001**	0.471	**0.016**	0.964	0.334
	Early sc.	0.169	**<0.001**	0.447	0.312	0.610	0.204
	Late sc.	0.504	**0.001**	0.528	0.085	0.834	0.593
RSR	Invasive	0.161	**<0.001**	**0.012**	0.390	0.186	0.178
	Early sc.	0. 962	**0.018**	0.762	0.461	0.588	0.097
	Late sc.	**0. 015**	**<0.001**	0.066	**0. 016**	0.680	0.849
TLA	Invasive	0.065	**0.007**	**0.017**	0.084	0.559	0.215
	Early sc.	0.156	**0.001**	0.297	0.067	0.770	0.898
	Late sc.	**0.017**	**0.001**	**<0.001**	**0.021**	0.127	0.099
SLA	Invasive	**< 0.001**	0.168	0.115	0.249	**0.023**	**<0.001**
	Early sc.	**< 0.001**	**0.020**	0.253	0.976	0.720	0.776
	Late sc.	**0.001**	0.052	0.655	0.165	0.988	0.593
LWR	Invasive	**0.012**	**<0.001**	**<0.001**	**0.021**	0.383	0.965
	Early sc.	0.184	**<0.001**	0.077	0.268	0.965	0.801
A_max_	Late sc.	0.437	**<0.001**	**<0.001**	**0.034**	0.784	0.349
	Invasive	0.710	0.043	0.168	0.450	0.897	0.986
	Early sc.	0.110	0.407	**0.041**	0.600	0.573	0.417
	Late sc.	0.588	**0.005**	**0.008**	0.055	0.243	0.553
A_maxw_	Invasive	**0.023**	0.086	0.130	0.242	0.541	0.752
	Early sc.	**0.002**	0.800	0.512	0.771	0.986	0.947
	Late sc.	0.098	**0.004**	0.095	**0.013**	0.457	0.243
N_m_	Invasive	0.836	**<0.001**	**0.012**	**0.008**	0.219	0.141
	Early sc.	0.603	**<0.001**	0.459	**0.002**	0.101	0.371
	Late sc.	**0.037**	**<0.001**	0.972	**<0.001**	0.773	0.548
PNUE	Invasive	0.253	0.213	0.171	0.398	0.048	0.107
	Early sc.	0.629	0.090	**0.029**	0.257	0.634	0.213
	Late sc.	0.056	**0.037**	**0.023**	0.120	0.777	0.426
N_a_	Invasive	**<0.001**	**0.006**	**0.018**	**0.007**	0.445	0.957
	Early sc.	**<0.001**	**<0.001**	0.479	**<0.001**	**0.028**	0.374
	Late sc.	**<0.001**	**<0.001**	0.872	**<0.001**	0.812	0.654

Significant p values (p < 0.05) are presented in bold. Species are grouped by strategy: the invasive species is *Acer negundo*, early-successional native species are *Salix alba *and *Populus nigra *and late-successional native species are *Fraxinus excelsior *and *Fraxinus angustifolia*. Traits are: RGR_h _relative growth rate in seedling height, RSR root/shoot ratio, TLA total leaf area, SLA specific leaf area, LWR leaf weight ratio, A_max _light-saturated assimilation rate per unit leaf area, A_maxw _light-saturated assimilation rate per unit leaf dry weight, N_m _nitrogen content, N_a _nitrogen content per unit leaf area and PNUE the photosynthetic nitrogen use efficiency.

### Biomass allocation and specific leaf area

**Figure 2:**
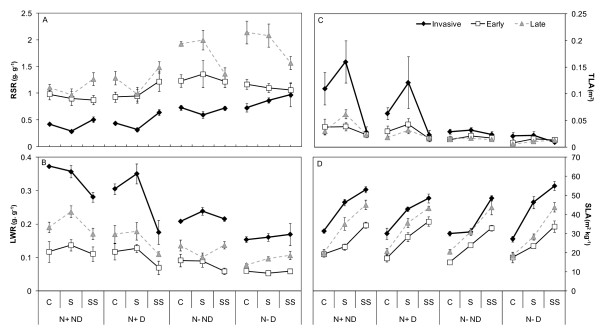
**(A) Root/shoot ratio, (B) leaf weight ratio, (C) total leaf area and (D) specific leaf area of the invasive and native species according to the environmental conditions**. Root/shoot ratio (RSR), total leaf area (TLA, m^2^), leaf weight ratio (LWR, g. g^-1^), specific leaf area (SLA, m^2^. kg^-1^). Values are means of nine to twelve seedlings (± 1 SE of the mean) for the invasive species (*A. negundo*, full diamonds), late-successional native species (*F. excelsior *and *F. angustifolia*, grey triangles) and early-successional native species (*S. alba *and *P. nigra*, open squares) across the three shade levels (Full light C, Shade S, Deep shade SS), the two nutrient levels (nutrient supply N+ vs. no supply N-) and the two disturbance regimes (Disturbed D vs. Non-disturbed ND).

Overall, nutrient availability was the main factor affecting biomass allocation, the response to light availability being trait and species dependent. Allocation to roots was significantly lower under the fertilised treatments (Table 1), with a 1.8, 1.2 and 1.6 reduction for the invasive, early- and late-successional species, respectively. The LWR increased with fertilisation for all species (Figure 2). However, for the invasive species, responses to fertilisation in allocation towards foliage were primarily significant under the fertilised full light and shade treatments only (significant shade*fertilisation p = 0.0213 on LWR, Table 1). TLA was significantly increased by fertilisation for all species (Table 1): for the invasive, TLA was 3.7 times higher compared to non-fertilised treatments, vs. only 2.1 and 2.3 times higher for the early-and late-successional species. The invasive species displayed a lower RSR than the native species under fertilised conditions whatever the light treatment (0.01 < p < 0.05, Figure 2). Late-successional native species presented the highest allocation to roots and significant differences in allocation to roots in response to light availability (shade p = 0.0148, shade*fertilisation p = 0.0161, Table 1, Figure 2) with a fertilisation interaction. The invasive species also presented a higher allocation to leaves than the native species across all treatments (0.0003 < p < 0.02; +170 and +74% increase in mean LWR, compared to the native early- and late-successional species, respectively). Under fertilisation and full light or shade conditions, the TLA of the invasive species reached three-fold higher values than either early- and late-successional species (p < 0.01), similarly to that observed for relative growth rate and allocation to foliage (Figure 2).

**Figure 3:**
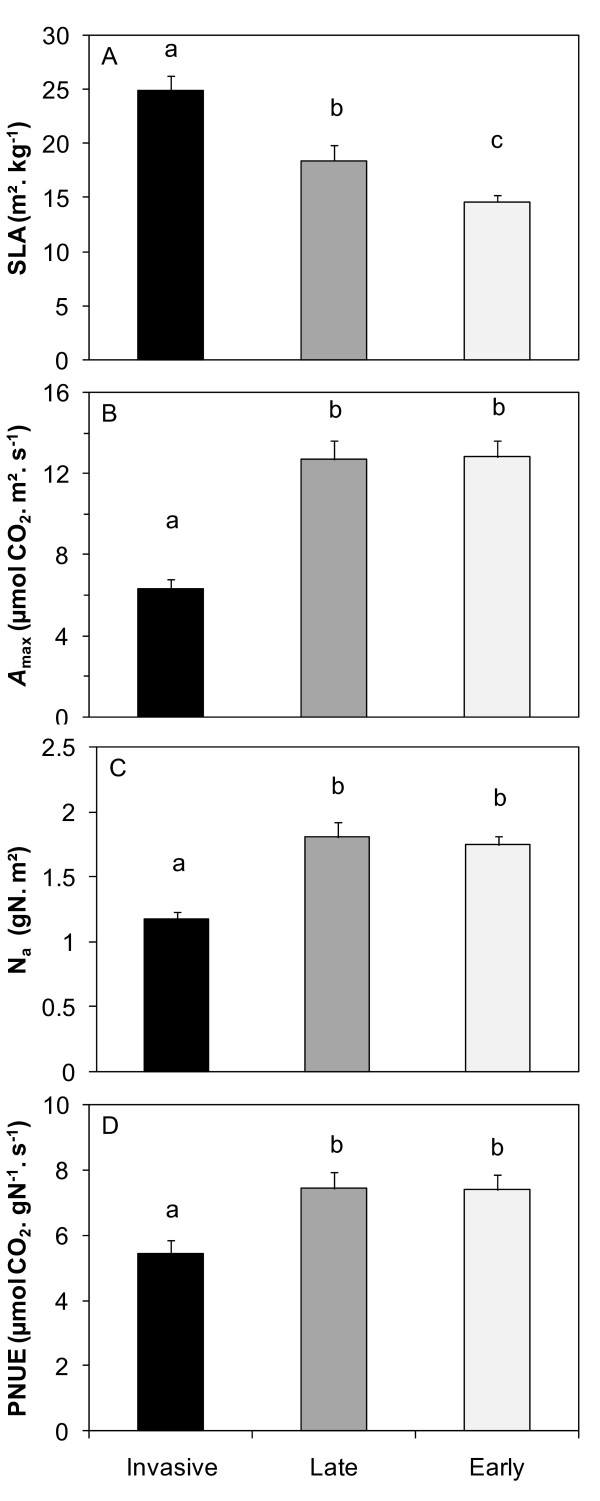
**(A) Specific leaf area, (B) light- saturated assimilation rate, (C) leaf nitrogen content and (D) photosynthetic nitrogen use efficiency of the invasive and native species in the field**. Specific leaf area (SLA, m^2^. kg^-1^), light-saturated assimilation rate (A_max_, μmol CO_2_. m^-2^. s^-1^), leaf nitrogen content (Na, g. m^-2 ^) and photosynthetic nitrogen use efficiency (PNUE, μmol CO_2_. g^-1 ^N. s^-1 ^) of the invasive species (*A. negundo*, full bars), the late-successional native species (*F. excelsior*, grey bars) and the early-successional native species (*A. glutinosa*, light-grey bars) as measured *in situ*. Values are means of 25 to 34 adult trees (± 1 SE of the mean). ANOVA were highly significant for all variables, respectively: F = 18.51 p < 0.0001; F = 26.85 p < 0.0001; F = 19.6 p < 0.0001; F = 6.96 p = 0.0016. Means with the same letters are not significantly different (α = 0.05).

All the species in the greenhouse experiment presented significantly lower SLA under increased light regimes (p < 0.001, Table 1), whereas fertilisation and disturbance had no effect. Furthermore, the invasive species seedlings exhibited higher SLA than the native ones, SLA values being 1.6 and 1.3 times higher on average for the invasive species compared to the early- and late-successional species, respectively (Figure 2, Additional file 1). *In situ *measurements on adult trees (Figure 3) indicated similar differences between species groups (p < 0.001), with higher SLA values for the invasive species compared to the native early- and late-successional ones (ratio 1.7 and 1.4, respectively, Additional file 1).

### Physiological traits

**Figure 4:**
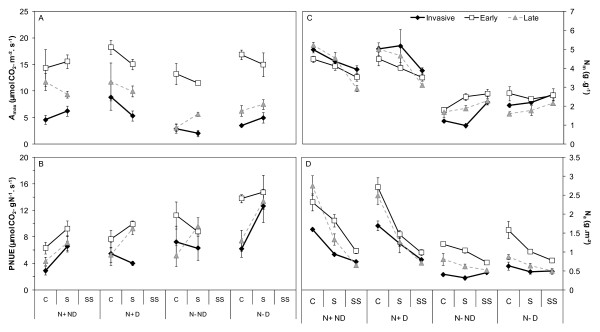
**(A) Light-saturated assimilation rate, (B) photosynthetic nitrogen use efficiency and (C, D) leaf nitrogen contents of the invasive and native species according to the environmental conditions**. Light-saturated assimilation rate (A_max_, μmol CO_2_. m^-2^. s^-1^), leaf nitrogen content (N_m _%, N_a _g. m^-2^) and photosynthetic nitrogen use efficiency (PNUE, μmol CO_2_. g^-1^N. s^-1^). Values are means of nine to twelve seedlings (± 1 SE of the mean) for the invasive species (*A. negundo*, full diamonds), late-successional native species (*F. excelsior *and *F. angustifolia*, grey triangles) and early-successional native species (*S. alba *and *P. nigra*, open squares) across the three shade levels (Full light C, Shade S, Deep shade SS), the two nutrient levels (nutrient supply N+ vs. no supply N-) and the two disturbance regimes (Disturbed D vs. Non-disturbed ND).

The same physiological traits - photosynthetic assimilation rate, leaf nitrogen content and photosynthetic nitrogen use efficiency - were measured on seedlings in the greenhouse (Figure 4) and on adult trees in the field (Figure 3). *A_max _*and *A_maxw _*were quite conservative over the different environments for all species, with no significant differences according to the shade, fertilisation or disturbance treatments (except a fertilisation effect for the late-successional native species, Table 1). The leaf nitrogen contents (N_m_, %) significantly increased with fertilisation, whatever the light availability and disturbance regime. The pattern observed in the response of nitrogen content on a leaf area basis (N_a_) to shade and fertilisation was similar for all species: N_a _significantly increased with fertilisation in interaction with the shade treatment, the nitrogen content being on average three times higher under full light * fertilisation treatment (Table 1, Figure 4), compared to the other modalities. Overall, the treatments had no significant effects on PNUE (Table 1).

The invasive tree species had significantly lower photosynthetic capacities (*A_max_*, *A_maxw_*) than both the native early- and late-successional species which performed equally. *In situ*, the light saturated assimilation rate of the invasive species equalled half that of the natives. The differences observed on the seedlings were quite similar, the early-successional species presenting the highest photosynthetic rates (species group effect: 0.01 < p < 0.04), from 1.5 to 5.7 time increase, according to the treatment; the invasive species performed equally to the late-successional natives (Figure 4). No difference was found between species in leaf nitrogen content expressed on a biomass basis (N_m_, Figure 4) whereas N_a _of the early-successional species was significantly higher compared to that of the late-successional and the invasive species (0.003 < p < 0.05, according to the treatment). In the field on adult trees, stronger differences were found, with both early- and late-successional species presenting higher nitrogen contents than the invasive species (p < 0.001; 70% more compared to the natives, Figure 3). On adult trees *in situ*, PNUE demonstrated the lower efficiency of the invasive species compared to the natives (p = 0.002, Figure 3); in the greenhouse, the photosynthetic nitrogen use efficiency was not significantly different between the species (Figure 4).

## Discussion

In the present study, we compared the growth, physiology and allocation patterns of an invasive tree species, *A. negundo*, to co-occurring native tree species across a wide range of controlled environmental conditions including light, nutrient availability and disturbance using 1 year-old seedlings under greenhouse conditions and adult trees in the field. Overall, *A. negundo *seedlings grew better under high-level resource environments (full light and fertilised). The relative success of *A. negundo *was, however, not related to any physiological advantage *per se *but to its higher plasticity in allocation to foliage in response to increasing nutrients and light.

### Functional strategies

We showed that under high resource environments, the invasive *A. negundo *exhibited higher growth than the co-occurring native tree species. This finding is consistent with a large majority of studies conducted on woody species wherein invasives outcompeted natives in the field [11,13,15,18,19] or in experimental plots [14,45,46]. Using a transplant design in the field, Saccone et al. [43] showed that *A. negundo *could outcompete native species through a trade-off between high survival in shaded environments and high growth under full light conditions. For species of the same genus *Acer*, Kloeppel and Abrams [12] demonstrated that the height growth increment of the native *A. saccharum *was more than two times lower than the growth rate of the invasive *A. platanoides*.

In our study, the growth success of the invasive tree was not related to any physiological advantage over its native counterparts. On the contrary, both in the field for adult trees and under all light and nutrient controlled conditions for seedlings, *A*. *negundo *photosynthetic rates and leaf nitrogen contents (N_a_, N_m_) were lower or equivalent to those measured on the late and early-successional native species. Several studies reported equivalent photosynthetic rates or characteristics (V_cmax_, J_max_, Fv/Fm) when comparing invasive and native tree species [15,18,20] or shrubs [47]. In some studies, a physiological advantage was even demonstrated in favour of the invasives [12,19,48]. No previous study on woody plants demonstated a physiological inferiority of the invasive species. In the literature regarding the nitrogen leaf content and nitrogen use efficiency, most studies concluded to a superiority of invasive tree species [13,15,20,49-51] and some to an absence of differences [12,47]. Again no similar study involving tree species ever demonstrated a net and significant physiological disadvantage related to nitrogen content of the invasive tree compared to its local native competitors. Thus although we have been working on seedlings, the findings of our study are novel for they represent the first study on woody plants to our knowledge that demonstrated that the growth superiority of an invasive tree was associated to a physiological disadvantage relative to the natives; such a paradox has only been observed one time out of four on herbaceous species (review by [25]).

The specific allometric properties of *A. negundo *clearly demonstrated that despite its poor physiological performances, it could outcompete local species growth due to a large investment in the development of aerial structures (lower RSR, higher LWR and TLA, higher SLA) thus maximising solar radiation capture. Under controlled conditions, its total leaf area can represent up to three times that of the native seedlings, its leaf weight representing 20 to 40% of its total biomass, in opposition with the compared natives (5-20%). Large relative investment in foliage of invasive species compared to co-occurring natives was commonly observed [15,18,52,53]. However few studies really measured the biomass repartition between compartments of invasive tree species and SLA was more largely measured in trees as a proxy to detect higher light resource capture capacities. The higher SLA values that we observed in *A. negundo *were in accordance with many studies covering more than 50 species of woody invasives [13,15,18,47,49,50,53,54].

Our study also generally supports the conclusions of a recent synthesis comparing 34 woody species in Argentina, including the invasive *A. negundo *[54], which found that large leaf and foliage trait values (SLA and TLA) can be common characteristics to woody invasive species; but contrary to our conclusion, they also emphasized a physiological superiority as an explanation for invasiveness. Hence, this synthesis concluded that invasive and native woody species differ in functional strategies. Another synthesis recently published using the relationships between structural (SLA) and physiological trait values (A_max_, N content) concluded that native and invasive species (122 species in Australia) use similar strategies for light capture and carbon assimilation [55]; the success of invasive species was thus generated by their positions at the higher end of the range of species traits values. Similarly, Thompson and Davis [56] proposed to use a continuous scale of traits to compare species from "loser" to "winner" species; *A. negundo *would then be identified as a "winner" species. However, our results do not support these hypotheses since the native species physiological characteristics largely exceeded those of the invasive, whereas *A*. *negundo *clearly demonstrated a specific strategy of massive investment in leaf foliage, which largely compensated for its lower photosynthetic rates and nitrogen use efficiency. This strategy can explain its elevated growth rates under high resource environments and its invasiveness in riparian habitats.

### Magnitude of plasticity

Our experiment demonstrated that *A. negundo *is highly plastic in growth and traits such as TLA or LWR in response to changes in nutrient availability and light levels. *A. negundo *seedlings performed poorly relative to natives under low nutrient conditions whatever the light regime and under fertilised but light-limited environments. *A. negundo *also strongly benefited from increases in light and nutrients whereas native species plasticity remained limited. Indeed, it seems that the success of invaders relative to local species is highly dependent on the growing conditions [25], as the native species would stand up to the competition impeding invasion success under stressful environments (low nutrient, water or light availability). In accordance with our results, several studies also showed a pattern of superior allocation plasticity in invasive species and a massive investment to foliage in response to resource enrichment [8,15,21,50,52,53,57]. Very few studies examined the physiological-trait plasticity in invasive tree species. Nonetheless, three studies have found a higher plasticity in photosynthetic characteristics [19,21] and nitrogen content [50] in the natives with increasing resources compared to the invasives, while several others found a higher plasticity of invasive woody species in growth responses to nitrogen and/or light compared to the natives [14,19,46,49,58,59]. A recent experiment comparing invasive and native vines [53] concluded to the superior plasticity of the invasives in traits related to growth and allocation (LWR, SLA) and not in physiological traits (A_max_, WUE), in response to light availability, which is in total accordance with our conclusions. So both responses can occur in invaded forests, higher or lower plasticity of the invasive species, likely depending on the particular species and the characteristics of the invaded system. Our study forms a first comparison of native and invasive tree species that covers both field and controlled resource conditions, investigating physiology and allometry, which allowed us to increase our knowledge regarding the mechanisms of invasiveness of *A. negundo*.

In the conceptual framework of Richards's theory of plasticity [27] three strategies were proposed by which invaders can outcompete native species. (i) Jack-of-all-trade, the invader having superior abilities across stressful environments, (ii) Master-of-some, the invader being able to outcompete its counterparts under favourable conditions only and (iii) Jack-and-master a combination of both strategies. Our results clearly show that *A. negundo *has a master-of-some strategy that can explain the secret of its success at least in the riparian forests. Higher plasticity in allocation traits can allow *A. negundo *individuals to rapidly benefit from changes in their environmental conditions (nutrient availability, light) thereby capitalising on the fluctuating resources of these specific riparian ecosystems to overgrow local species. Thus, in the actual context of increasing nitrogen deposition [60], the spread of *A. negundo *could be accentuated due to both its greater performance under high nutrient availability and to its higher plasticity relative to native species. Dramatic impacts of nitrogen deposition on forest functioning have indeed been demonstrated, particularly the increase of the annual rate of biomass increment [61] and the facilitation of invasions [62].

## Conclusions

Our study added to the general debate on the mechanisms and species traits that explain the success of invasive tree species over their native counterparts. The success of *A. negundo *as an invasive species is likely to be driven by its superior growth ability compared to native species in resource-rich environments (light, nitrogen), due to a higher plasticity in biomass allocation. Moreover, two further steps would be particularly relevant to determine: (i) whether the higher magnitude of plasticity is adaptive by relating trait values to fitness proxies under different environments [59] and (ii) whether the invasive populations present genetic differentiation in the plasticity of their traits [10,63] by comparing populations from both the native and invasive ranges.

High plasticity in biomass allocation could be a key to understanding tree species invasiveness; the plastic response of *A. negundo *could impede its establishment under closed-canopy hardwood forests while its high plasticity would perfect its growth and potentially allow its spread in resource-rich riparian forests down to the river.

## Methods

### Studied species

Native to North America, *Acer negundo *L. is the most widely distributed of all North American maple. *A. negundo *was intentionally introduced in Europe during the seventeenth century (in France around 1749 [64,65]). It is a small to medium sized tree with pinnately compound leaves that usually have five leaflets. First planted in parks, this species is now widely used in South of Europe as an urban tree for avenues for ornamental purposes. The actual distribution area of *A. negundo *in Europe now extends from southern France to Lithuania and from Italy to Germany [66]. In France, its ongoing invasion takes place in the southern two-thirds [67], mainly in riparian habitats. This species is of limited commercial importance and is considered an ecological pest inducing biodiversity losses and river banks instability [68].

At the interface between aquatic and terrestrial ecosystems, riparian forests constitute a key ecosystem that shapes many species' habitats [69] and are particularly vulnerable to invasions [4]. *Acer negundo *mostly invades riparian zones at the ecotone between native softwood and hardwood communities [43,44,70]. In these habitats, five native species can commonly be found in France and thus are likely to compete one or two at a time with *A. negundo*: *Populus nigra*, *Salix alba *and *Alnus glutinosa *are early-successionnal species highly tolerant to disturbances; *Fraxinus excelsior *and *Fraxinus angustifolia *are late-successional and more shade-tolerant species.

### Greenhouse experiment design

The objective was to compare the invasive tree species, *A. negundo*, to the four native tree species: *F. excelsior*, *F. angustifolia*, *S. alba *and *P. nigra*. During fall 2003 seeds of *A. negundo *and both *Fraxinus *species were collected *in situ *on populations located along the Garonne River and were sown after vernalization, in spring 2004 at the nursery of the INRA Pierroton research station (44°44'N 0°46'W, west of Bordeaux, Gironde, France). In February 2005, one-year-old seedlings of *S. alba *and *P. nigra *were bought. In March 2005, seedlings of all five species were transplanted in 4 L pots filled with a commercial sphagnum soil mixture (organic matter 80 % of dry matter, pH = 6; Le terreau du producteur, HTA, Saint Cyr en Val, France) and placed in a greenhouse under natural air relative humidity and controlled temperature (day T° 25°C and night T° 15°C). Plants were watered daily to field capacity. The experiment was arranged in a split-split-plot design with complete random blocks (3). The treatments were applied to mimic riparian habitat conditions: shade (3 levels, main plot), nutrient availability (2 levels, sub-plot) and mechanical disturbance (2 levels, sub-sub-plot). Treatments were applied from April 1st 2005, 15 days after leaf unfolding, till June 14th. The shade treatments consisted in a control full light (C, 100% of the ambient radiation), shade (S, 25% of full light) and deep shade (SS, 7% of full light). It was obtained combining thermal cloths over the plants. The nutrition treatment was obtained by providing a complete fertiliser (N+, 4 mg of fertilizer Compo Floranid Permanent, 16% N; 7% P_2_O_5_; 22.5% SO_3_; + metal elements) versus no fertiliser (N-). The fertiliser was applied three times on the 3^rd^, 14^th ^and 53^rd ^day after the start of the experiment. The fertiliser treatment corresponded to a nutrient level equivalent to that of riparian forest soils in South-West France [71,72]. Finally, disturbance (D) by river bank flooding was simulated by applying a hand-made partial defoliation (25%, on the 21^st ^and 48^th ^day after the start of the experiment) and compared to non-disturbed (ND) plants. Four individuals per species were randomly assigned to each of the 12 treatments, leading to a total of 720 individuals.

### Growth and biomass measurements

At the beginning and at the end of the experiment, total height (cm, ruler, nearest mm, H1 and H2 respectively) was measured on each seedling. The relative height growth rate (RGR_h_, mm. mm^-1^.d^-1^) was calculated for each individual as the difference between the logarithms of final and initial height divided by the number of days between the beginning of the experiment and the harvest:

(1) $$RGR_{h}=\frac{ln\left(H_{2}\right)-ln\left(H_{1}\right)}{t_{2}-t_{1}}$$

where ln (*H*_1_) and ln (*H*_2_) are the ln-transformed plant heights at the initial (t_1_) and final (t_2_) time of the experiments respectively [73].

At the end of the experiment, all seedlings were harvested to measure above- and below-ground biomasses (oven-dried at 65°C until constant dry weight) which were used to calculate the root/shoot ratio (RSR, g.g^-1^). Within each treatment and block, 180 plants out of the 720 were sampled randomly but equally amongst the treatments and species to undertake detailed biomass measurements: leaves, stems (branches + stem) and roots were separated. All the leaves were immediately set in distilled water for a minimum of 12 h to reach full hydration [74] and total leaf area per individual (TLA, m^2^) was determined then with a planimeter (Light box, Gatehouse, Scientific Instruments LTD, Norfolk, UK). Stem, root and leaf dry weights (oven-dried at 65°C until constant weight) were measured. For each species, specific leaf area (SLA, m^2^.kg^-1^) was calculated as the ratio of TLA to leaf dry weight; the leaf weight ratio (LWR, g.g^-1^) as the ratio of leaf dry weight to total individual biomass (stems + leaves + roots).

### Photosynthesis and nitrogen content measurements

Gas exchange measurements were carried out in early June, between 8.00 am and 12.00 am, with a steady state through flow chamber (PLC4, PP-Systems, Hitchin, UK) coupled with an infra-red gas analyzer (CIRAS II, PP-Systems, Hitchin, UK). During the measurements, air CO_2 _concentration, air temperature and relative humidity (RH) in the chamber were controlled to match ambient air values: 375 ± 3 ppm of CO_2_, 25 ± 1°C and 70 ± 10% of RH. All the measurements were made at saturated light (PPFD = 1500 μmol.m^-2^.s^-1^) in order to obtain a light-saturated photosynthetic assimilation rate (A_max_, μmol CO_2_.m^-2^.s^-1^) at ambient CO_2_. No gas exchange measurements were conducted under the deep shade treatment due to the very low number of leaves per individual. For *Salix alba*, no measurements could be performed either, whatever the treatment, due to a too small leaf size compared to the leaf chamber surface. Three repetitions were made per species and per treatment, leading to a total number of 96 photosynthesis measurements. Light-saturated photosynthetic assimilation rate per unit leaf dry weight (A_maxw_, μmol CO_2_.kg^-1^.s^-1^) was calculated as the ratio of A_max _to SLA.

Leaf nitrogen content was analysed from the leaf samples used for photosynthetic rate measurements (n = 96). Leaf samples were crushed to powder with a ball mill (MM 200, Fisher Bioblock Scientific, France), then nitrogen content (N_m_, %) was measured with an elementary analyser Eager 300 CHONS (FlashEA 1112, ThermoElectron Corporation, Waltham, MA, USA). Nitrogen content per leaf area (N_a_, g N.m^-2^) was calculated as N_m _divided by SLA and the photosynthetic nitrogen use efficiency (PNUE, μmol CO_2_.g N^-1^.s^-1^) as A_max_/ N_a_.

### *In situ *measurements

*In situ *measurements were conducted in May 2006 in four invaded riparian habitats of South-West France. Two sites were located in Cestas along the Eau Bourde River (44°45'20.37''N, 0°40'49.95''W and 44°44'47.00''N, 0°41'17.93''W), one in Bruges along The Jalles River (44°54'12.45''N, 0°36'16.40''W) and one in Saint-Denis-de-Pile along the Isle River (44°59'35.66''N, 0°12'28.45''W). In each site, ten adult individuals from the upper canopy were selected for each species (the invasive species *A. negundo *and the co-occurring native species late-successional *F. excelsior *and early-successional *Alnus glutinosa*). Light-saturated photosynthetic assimilation rate measurements were carried out following the same protocol as for the greenhouse experiment. Leaves used for photosynthesis measurements were collected and their leaf area, dry weight, SLA and nitrogen contents were determined as indicated previously.

### Statistical analyses

Statistical analyses were conducted using the SAS software package (SAS 9.1, SAS Institute Inc., Cary, NC). For the controlled conditions experiment, a split-split-plot analysis of variance was performed (proc GLM) and mean differences assessed with SNK and Tukey multiple comparison tests (α < 5%). Main plot (shade) and block effects were tested using shade*block as an error term, the sub-plot effects (fertilisation, fertilisation*shade) were tested using block*fertilisation(shade) as an error term and sub-sub-plot effects (disturbance, disturbance*shade, disturbance*fertilisation, disturbance*shade*fertilisation) were tested using the regular error term according to Federer and King [75,75]. Analysis of variance (proc GLM) and SNK multiple comparison tests (α < 5%) were used to test species differences *in situ*.

## Authors' contributions

AJP developed the theoretical background and drafted the manuscript. LJL collected the data, contributed to the data analysis and drafted the manuscript. CJL contributed to the theoretical background and revised the manuscript. RM was at the initiative of the project, contributed to the funding and data analysis. SD constructed the experimental design, carried out the statistical analysis and drafted the manuscript. All authors read and approved the final manuscript.

## Supplementary Material

Additional file 1 **Means and Tukey groups per species group for all measured traits and tested experimental conditions**. For a given trait different letters on the same column indicate significant differences amongst species groups for a combination of light, fertilisation and disturbance (Tukey test). Species are grouped by strategy: the invasive species is *Acer negundo*. Native early-successional species are *Salix alba *and *Populus nigra*, and native late-successional species are *Fraxinus excelsior *and *Fraxinus angustifolia*. Traits are RGR_h _relative height growth rate (mm. mm^-1^.d^-1^.10^-3^), RSR root shoot ratio (g. g^-1^), TLA total leaf area (m^2^), SLA specific leaf area (m^2^. kg^-1^), LWR leaf weight ratio (g. g^-1^), A_max _light-saturated assimilation rate (μmol CO_2_. m^-2^. s^-1^), N_m _nitrogen content (%), N_a _leaf nitrogen content (g. m^-2^)and PNUE photosynthetic nitrogen use efficiency (μmol CO_2_. g^-1^N. s^-1^). Environmental conditions are: Fertilised (N+), Non-fertilised (N-), Disturbed (D), Non-disturbed (ND), Full light (C), Shade (S) and Deep shade (SS). Click here for file
