# Supplementary material for: Invasive Acer negundo outperforms native species in non-limiting resource environments due to its higher phenotypic plasticity
Source: BMC Ecol. 2011 Nov 24;11:28. doi: 10.1186/1472-6785-11-28 (PMC3275484; doi:10.1186/1472-6785-11-28)
Supplement: Additional file 1 — Means and Tukey groups per species group for all measured traits and tested experimental conditions. For a given trait different letters on the same column indicate significant differences amongst species groups for a combination of light, fertilisation and disturbance (Tukey test). Species are grouped by strategy: the invasive species is Acer negundo. Native early-successional species are Salix alba and Populus nigra, and native late-successional species are Fraxinus excelsior and Fraxinus angustifolia. Traits are RGRh relative height growth rate (mm. mm-1.d-1.10-3), RSR root shoot ratio (g. g-1), TLA total leaf area (m2), SLA specific leaf area (m2. kg-1), LWR leaf weight ratio (g. g-1), Amax light-saturated assimilation rate (μmol CO2. m-2. s-1), Nm nitrogen content (%), Na leaf nitrogen content (g. m-2)and PNUE photosynthetic nitrogen use efficiency (μmol CO2. g-1N. s-1). Environmental conditions are: Fertilised (N+), Non-fertilised (N-), Disturbed (D), Non-disturbed (ND), Full light (C), Shade (S) and Deep shade (SS). [file 1472-6785-11-28-S1.PDF]

Tukey grouping		N+						N-					
		ND			D			ND			D		
Variables	Species	C	S	SS	C	S	SS	C	S	SS	C	S	SS
RGR_h	Invasive	7.22a	8.45a	0.52b	5.87a	7.68a	0.64b	2.23a	1.64b	0.73a	3.11a	1.55b	0.24a
	Early sc.	5.90a	6.80ab	5.81a	5.05ab	6.84a	4.15a	3.55a	3.8a	2.8a	3.34a	4.28a	3.16a
	Late sc.	3.43b	4.99b	2.91b	2.97b	4.54b	2.44ab	1.35a	1.69b	2.21a	1.72a	1.92b	1.51a
RSR	Invasive	0.42b	0.29b	0.51c	0.44b	0.32b	0.64b	0.73c	0.59b	0.72b	0.73b	0.86b	0.97b
	Early sc.	0.98a	0.90a	0.87b	0.93a	0.95a	1.22a	1.23b	1.36a	1.22a	1.17b	1.10b	1.06b
	Late sc.	1.11a	0.97a	1.27a	1.29a	0.99a	1.48a	1.93a	1.99a	1.36a	2.14a	2.08a	1.57a
TLA	Invasive	109.8a	160.1a	28.1a	63.4a	121.2a	23.1a	29.2a	32.3a	23.5a	20.9a	21.8a	9.5a
	Early sc.	37.8b	38.3b	23.3a	29.0b	42.6b	16.0a	14.1b	20.6b	17.0a	7.9b	15.8a	13.1a
	Late sc.	29.7b	62.0b	23.0a	18.2b	31.8b	17.0a	17.4b	16.6b	14.4a	5.9b	10.5a	13.2a
SLA	Invasive	31.19a	46.29a	53.05a	30.02a	42.74a	48.50a	30.02a	30.85a	48.54a	27.12a	46.36a	55.00a
	Early sc.	19.33b	23.03c	34.43c	17.04b	28.12b	36.37b	15.06c	24.01b	32.85b	17.58b	23.50b	33.70c
	Late sc.	19.82b	35.09b	45.10b	20.63b	35.64ab	43.26a	20.60b	31.02a	43.75a	18.52b	28.33b	43.81b
LWR	Invasive	0.37a	0.36a	0.28a	0.31a	0.35a	0.17a	0.21a	0.24a	0.21a	0.15a	0.16a	0.17a
	Early sc.	0.12b	0.14c	0.11b	0.12b	0.13b	0.07b	0.09c	0.09b	0.06c	0.06b	0.05c	0.06b
	Late sc.	0.19b	0.24b	0.17b	0.17b	0.18b	0.11b	0.14b	0.10b	0.14b	0.08b	0.10b	0.11b
A_{max}	Invasive	4.44a	5.78b		8.87a	5.33c		2.92b	2.03c		3.41b	4.98b	
	Early sc.	13.64a	15.35a		18.26a	15.04a		13.23a	11.55a		16.83a	13.86a	
	Late sc.	12.17a	9.38b		11.19a	9.95b		3.11b	5.64b		6.26b	7.51b	
A_{maxw}	Invasive	0.15a	0.13b		0.29a	0.13a		0.10a	0.07b		0.14b	0.13b	
	Early sc.	0.79a	0.66a		1.06a	0.58a		0.98a	0.49a		0.91a	0.66a	
	Late sc.	0.63a	0.28b		0.52a	0.30a		0.16a	0.19b		0.36b	0.28b	
N_m	Invasive	4.99ab	4.36a	3.94a	5.03a	5.19a	3.87a	1.23a	0.97c	2.21a	2.08a	2.2a	2.61a
	Early sc.	4.48b	4.11a	3.52ab	4.50a	4.01a	3.52ab	1.81a	2.49a	2.80a	2.67a	2.38a	2.56a
	Late sc.	5.24a	4.54a	2.93b	5.02a	4.66a	3.11b	1.67a	1.89b	2.30a	1.60a	1.77a	2.17a
PNUE	Invasive	2.92a	6.58a		5.46a	4.01b		7.23a	6.28b		6.19a	12.66a	
	Early sc.	6.33a	9.24a		7.66a	9.92a		11.25a	8.80a		13.78a	14.75a	
	Late sc.	4.34a	7.10a		5.28a	9.22b		5.16a	9.60a		7.42a	13.41a	
N_a	Invasive	1.60b	0.94b	0.74b	1.70b	1.22a	0.80a	0.41c	0.32c	0.46c	0.63b	0.48b	0.50b
	Early sc.	2.32ab	1.83a	1.03a	2.72a	1.47a	0.99a	1.21a	1.04a	0.73a	1.59a	1.02a	0.78a
	Late sc.	2.76a	1.34ab	0.65b	2.50a	1.27a	0.72a	0.81b	0.63b	0.52b	0.88b	0.65b	0.50b
